# Self-Assembly of Hydrophobic Hyperbranched PLMA Homopolymer with –COOH End Groups as Effective Nanocarriers for Bioimaging Applications

**DOI:** 10.3390/polym16152166

**Published:** 2024-07-30

**Authors:** Angelica Maria Gerardos, Aleksander Foryś, Barbara Trzebicka, Stergios Pispas

**Affiliations:** 1 Theoretical and Physical Chemistry Institute, National Hellenic Research Foundation, 48 Vassileos Constantinou Avenue, 11635 Athens, Greece; amgerar@eie.gr; 2 Department of Chemistry, National and Kapodistrian University of Athens, Panepistimiopolis, Zografou, 15771 Athens, Greece; 3 Centre of Polymer and Carbon Materials, Polish Academy of Sciences, 34 ul. M. Curie-Skłodowskiej, 41-819 Zabrze, Poland; aforys@cmpw-pan.pl (A.F.); btrzebicka@cmpw-pan.pl (B.T.)

**Keywords:** hyperbranched, polymers, polyelectrolytes, RAFT, nanoparticles, bioimaging

## Abstract

Nanomedicine is a discipline of medicine that applies all aspects of nanotechnology strategies and concepts for treatment and screening possibilities. Synthetic polymer nanostructures are among the many nanomedicine formulations frequently studied for their potential as vectors. Bioimaging is a valuable diagnostic tool, thus, there is always a demand for new excipients/nanocarriers. In this study, hydrophobic hyperbranched poly(lauryl methacrylate) (PLMA) homopolymers comprised of highly hydrophobic LMA moieties with –COOH polar end groups were synthesized by employing reversible addition-fragmentation chain transfer (RAFT) polymerization. Ethylene glycol dimethacrylate (EGDMA) was utilized as the branching agent. End groups are incorporated through the RAFT agent utilized. The resulting amphiphilic hyperbranched polymer was molecularly characterized by size exclusion chromatography (SEC), Fourier transformation infrared spectroscopy (FT–IR), and ^1^H–NMR spectroscopy. Pyrene, curcumin, and IR-1048 dye were hydrophobic payload molecules successfully encapsulated to show how adaptable these homopolymer nanoparticles (prepared by nanoprecipitation in water) are as dye nanocarriers. This study demonstrates a simple way of producing excipients by generating polymeric nanoparticles from an amphiphilic, hyperbranched, hydrophobic homopolymer, with a low fraction of polar end groups, for bioimaging purposes.

## 1. Introduction

The revolutionary blend of nanotechnology and medicine has paved the way for the multifaceted field of nanomedicine, offering opportunities to enhance the quality of healthcare and economically revolutionize the medical landscape [1,2,3]. Although conventional drugs continue to be the primary focus of drug research, there has been a growing trend toward the use of nanovectors. Nanoformulations primarily generate superior outcomes than standard un-complexed drugs due to targeting capabilities, increased surface area, and simple tunability. This is because most of the human body’s physiological processes occur at the nanoscale, and crucial biomolecules like proteins and antibodies are found in this range [3,4]. Nanoparticles are a versatile and powerful tool in modern medicine. Multiple forms such as polymeric nanostructures, liposomes, solid nanoparticles, nanoemulsions, and carbon-based nanoparticles can be beneficial in therapy and diagnosis [5]. The European Union’s investment of over 550 million euros in nanomedicine research and development projects is a testament to the potential of this ever-evolving field [6].

Polymeric nanoparticles have grown in popularity due to the many advantages they offer such as sustained drug release, active targeting, stimuli-sensitivity, and high drug encapsulation efficiency [7]. The evolution of advanced multifunctional polymeric nanoparticles with reliable control over structural and surface properties of nanostructures has been made possible by breakthroughs in controlled radical polymerization [8]. Solid polymeric nanoparticles, micelles, polymer conjugates, dendrimers, polymersomes, polyplexes, and lipomers (lipid-polymer hybrids) are examples of the various forms and sizes of polymer-based nanostructures [9]. Hyperbranched polymers are an excellent candidate for such nanostructures. In applications that do not demand structural precision, these polymers tend to prevail in the commercial sector over dendrimers. The production of dendrimers is labor-intensive and requires multiple steps and significant synthetic effort to achieve. These highly branched structures have a high end-group concentration, which offers several advantages like functionality and solubility [10,11,12]. The synthesis of hyperbranched macromolecules is easily achievable by implementing a chemical crosslinker in conjunction with RAFT polymerization. This form of polymerization has proven to be a highly effective and reliable approach, prevailing as the favored method among researchers seeking to produce architecturally intricate macromolecular structures [13]. In such instances, ethylene glycol dimethacrylate (EGDMA) has frequently been used as a branching agent [14,15]. RAFT represents a controlled radical polymerization technique that offers a practical solution for obtaining copolymers with predetermined lengths, copolymer compositions, and functional groups. Such a method results in complex structures that exhibit low values of dispersity [16,17,18]. Lauryl methacrylate is a monomer that exhibits hydrophobic properties [19]. It is considered a “biobased” acrylate monomer, as it can be synthesized by reacting methacryloyl chloride and lauryl alcohol, the latter of which is derived from plant fatty acids. This makes it an environmentally friendly option compared to some other synthetic monomers. Furthermore, high flexibility is provided by PLMA extended alkyl backbone, setting it apart from other acrylate polymers like methyl methacrylate [20,21,22].

Micelles and vesicles are supramolecular structures formed by spontaneous self-assembly of amphiphilic molecules. Amphiphilic polymers follow the same concept as single low-molar mass organic molecules. Nevertheless, research has demonstrated that amphiphilic macrosurfactants are more efficient when applied than low molecular weight surfactants, since most of them have much lower diffusion coefficients and critical micelle concentrations [23,24].

Polymeric nanoparticles are formed in aqueous solutions when the concentration exceeds a certain value known as the critical aggregation concentration (CAC). This occurs due to several synergistic effects, where hydrophobic moieties of copolymers can assemble to enclose dissoluble molecules. The structure is further stabilized due to the hydrophobic effect and/or electrostatic interactions. In this formation, the precious cargo is protected by the hydrophilic moieties, which form an outer layer that acts as a barrier. These structures offer numerous benefits such as improved solubility, higher bioavailability, and greater stability in biological media and storage conditions. More importantly, targeting abilities can be attained through the utilization of the enhanced permeability and retention (EPR) effect [25,26,27]. This process occurs as a result of gaps developing in the endothelial lining of malignant tumors, which facilitates the passage of small-sized vectors [28].

Curcumin (CUR), a phenolic compound, has been widely recognized for its medicinal properties for many years and is still at the forefront of research [29]. In 2023 alone, more than 56,000 papers featuring curcumin (source: Google Scholar) were published. Anti-inflammatory and antioxidant properties are its primary benefits. Curcumin is a less hazardous substitute for conventional imaging agents like commercial synthetic dyes and quantum dots, rendering it an excellent prospect for bioimaging applications. However, it should be noted that curcumin is photosensitive and limitations regarding its solubility consequently result in poor gastrointestinal absorption, rapid renal excretion, minimal bloodstream stability, and poor bioavailability. These challenges further decrease its efficacy. With every factor considered, curcumin’s distinct qualities make it a viable option for bioimaging applications when toxicity is a concern [30,31,32].

Traditional probes in photothermal therapy are known to cause high phototoxicity and have limited tissue penetration depth due to their excitation in the UV–Vis spectrum. Nevertheless, second near-infrared region (NIR-II) laser dyes have emerged as a novel solution. NIR light has a longer wavelength, and therefore a deeper penetration depth, which makes it an ideal choice for imaging deep-tissue structures. Despite the benefits of NIR-II laser dyes, it is worth noting that not all dyes are made equal. For instance, cyanine dyes such as IR-1048 have solubility and stability issues, which can limit their effectiveness in certain applications [33,34].

This article delves into the synthesis of a hyperbranched polymer through LMA polymerization, utilizing EGDMA as a branching agent via RAFT. In a subsequent stage, the homopolymer is utilized as self-assembled nanoparticles in aqueous media to encapsulate hydrophobic dyes, ultimately amplifying their efficacy, and highlighting the impressive capabilities of the resulting H-(PLMA) nanoparticles through a simple nanoprecipitation process.

## 2. Materials and Methods

### 2.1. Materials

LMA, monomethyl ether hydroquinone inhibitor remover, 2,2 azobisisobutyronitrile (AIBN), 4-cyano-4-(phenyl-carbonothioylthio)-pentanoic acid (CPAD), pyrene, Tween 80, curcumin, IR-1048, and all solvents including 1,4-dioxane (99.8% pure), tetrahydrofuran (THF), ethanol, deuterated chloroform (CDCl_3_) were supplied by Sigma Aldrich (St. Louis, Missouri, United States). EGDMA monomer, utilized as the branching agent, was purchased from Merck (Darmstadt, Germany). Water for injection (WFI) was purchased from DEMO AΒΕΕ (Attica, Greece) while fetal bovine serum (FBS) was from Gibco™ Invitrogen™ (Waltham, Massachusetts, USA ). The monomer was purified using a column packed with hydroquinone monomethyl ether inhibitor remover. All solvents used were analytical grade.

#### 2.1.1. Synthesis of H-(PLMA) and Linear PLMA

**Figure 1:**
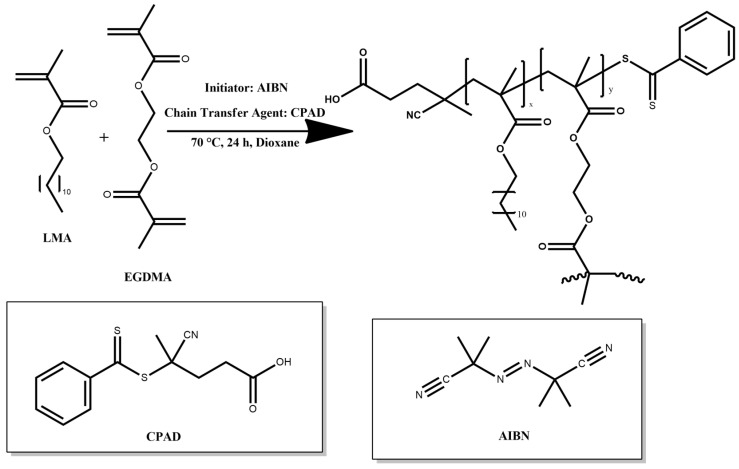
Illustration of the synthetic route for preparing H-(PLMA) homopolymer.

The hyperbranched homopolymer was synthesized via RAFT polymerization utilizing EGDMA monomer as the branching agent. AIBN was used as the radical initiator with CPAD as the chain transfer agent (CTA), and 1,4-dioxane was the selected solvent for the reaction (Figure 1). A typical protocol used for the synthesis of H-(PLMA) was as follows: in a round bottom flask, AIBN (32.842 mg, 0.2 mmol), CPAD (112 mg, 0.4 mmol), EGDMA (0.091 mL, 0.48 mmol), LMA (2 g, 7.861 mmol), and 7 mL of 1,4-dioxane was added. The contents of the flask were then stirred vigorously to ensure thorough mixing utilizing a magnetic stirrer. Following the homogenization of the mixture, nitrogen was then bubbled at room temperature for 20 min to deaerate the solution. Subsequently, the flask was immersed in an oil bath at 70 °C under agitation. The polymerization was allowed to proceed for 24 h and then was exposed to a −20 °C environment until frozen. The reaction was further quenched by exposing the reaction solution to air for the completion of the polymerization process. The product was precipitated in tenfold cold methanol to remove unreacted monomers. The precipitate was finally collected using THF and dried in a vacuum oven for 72 h at ambient temperature. The linear analog was produced using the same procedure except for the addition of the branching agent. The obtained homopolymers were characterized at the molecular level by SEC (Table 1), ^1^H–NMR, and FT–IR spectroscopy.

#### 2.1.2. Preparation of Neat and Dye-Loaded Nanostructures

Nanoparticles were generated using the nanoprecipitation technique. This approach involves violently dispersing an organic solution of the polymer in an aqueous medium, followed by slow displacement of the organic solvent through heating. The polymer swiftly diffuses into the aqueous phase, leading to the immediate formation of nanoparticles as a result of hydrophobic interactions. These interactions involve nonpolar groups such as alkyl groups, which tend to aggregate when exposed to a water medium [35]. In more detail, the homopolymer was dissolved in THF while the dye was dissolved in THF (CUR) or ethanol (IR-1048). The organic phases were then mixed and injected dropwise via a syringe into the aqueous phase under stirring, resulting in self-assembly. The samples were heated above the boiling point of the organic solvent with the use of a hot plate, leading to slow evaporation of the organic phase.

#### 2.1.3. Preparation of Mixed Systems

Tween 80 is a non-ionic surfactant that is frequently added as a solubilizing agent to prevent nanoparticles from clustering. The evaporation method was implemented with varying surfactant concentrations and dispersion methods. The homopolymer concentration was 10^-4^ g/ml. The mixed systems were prepared in weight ratios 1:1 (A series) and 1:0.3 (B series). The homopolymer was dissolved in THF and Tween 80 was subsequently incorporated, either dissolved in THF (TA/TB sample codes) or water (TWA/TWB sample codes). This subtle variation in the preparation process may have a significant impact on the properties of the resulting nanoparticles. Nanoparticle solutions for Dynamic light scattering (DLS) measurements were diluted to a factor of 3 before analysis.

### 2.2. Methods

#### 2.2.1. Size Exclusion Chromatography

A Waters system, consisting of a Waters 1515 isocratic pump, three µ-Styragel mixed bed columns with pore diameters ranging from 102 to 106 Å, and a Waters 2414 refractive index detector (equilibrated at 40 °C), was used to determine the molecular weight and molecular weight distributions of each sample. The eluent was THF set to a flow rate of 1.0 mL/min. The column set was calibrated using linear monodisperse polystyrene standards, with average molecular weights ranging from 1200 g mol^−1^ to 152,000 g mol^−1^. The data was collected and analyzed using the Waters Breeze software (Breeze v2.0 Waters Corporation, Milford, MA, USA).

#### 2.2.2. Proton Nuclear Magnetic Resonance Spectroscopy (^1^H–NMR)

A Varian 300 (300 MHz) spectrometer was employed, and the Vjnmr software (VNMRJ 2.2C, Varian, Palo Alto, CA, USA) was used for spectra acquisition. The solvent utilized to prepare the sample was CDCl_3_ (polymer concentration = 1-4 mg/mL). Chemical shifts are expressed in parts per million (ppm) with tetramethylsilane serving as an internal reference. MestReNova software (MestReNova 14.0.0, Mestrelab Solutions, Bajo, Spain) was used to analyze the obtained spectra.

#### 2.2.3. Fourier Transform Infrared Spectroscopy (FT–IR)

The measurements were carried out using a Fourier transform instrument (Bruker Equinox 55, Bruker Optics GmbH) fitted with a single bounce attenuated total reflectance (ATR) diamond accessory (Dura-Samp1IR II by SensIR Technologies). Spectra is the result of the average of 100 scans collected at 2 cm^−1^ resolution. Polymers were measured in solid form using a press.

#### 2.2.4. Dynamic Light Scattering

The average hydrodynamic radius (R_h_) and size distribution were measured by an ALV/CGS-3 Compact Goniometer System. The measurements were accomplished through the use of a JDS Uniphase 22 mW He–Ne laser (632.8 nm) at a fixed measuring angle of 90° at room temperature. The system was coupled to a digital ALV-5000/EPP multi-tau correlator with 288 channels. The autocorrelation functions were averaged five times within thirty seconds and were analyzed using the cumulants method and the CONTIN algorithm.

##### Suspension of Nanoparticles in Fetal Bovine Serum

The basis of this method is to evaluate in a first step the biocompatibility of novel nanoparticles. When exposed to FBS polymer-based nanoparticles tend to aggregate. According to proteomic studies, this serum contains more than 1800 distinct proteins. [36]. The most abundant of these proteins, bovine serum albumin (BSA), makes up approximately 50% to 60% of the total serum protein and is present at a concentration of roughly 2.5 mg/mL [37,38]. As a result of its widespread availability, FBS is frequently used in nanomedicine research to replicate the physiological conditions that nanoparticles might encounter in the bloodstream. Whether the serum triggers the formation of aggregates or a protein corona and changes the surface chemistry of the nanoparticles was examined. The dilution factor of FBS to WFI was a 1:1 volume ratio and maintained a 1:1 ratio of homopolymer to protein suspension.

#### 2.2.5. Electrophoretic Light Scattering

A Malvern system (Nano Zeta Sizer) with a 4 mW He–Ne laser with a wavelength of 633 nm was used to acquire the measurements. The average of 100 consecutive scans conducted at ambient temperature is represented by each value reported here.

#### 2.2.6. UV/Vis/NIR Spectroscopy

The UV/Vis/NIR spectra were recorded in the 200–600 nm (CUR) and 700–1100 nm (IR-1048) regions using a Perkin Elmer Lambda 19 UV–Vis–NIR spectrometer. The scan speed was adjusted to 960 (IR-1048) and 240 (CUR) scans/s per measurement. Given the use of a double-beam spectrometer, a reference cuvette containing the dispersion medium was utilized as the reference for all measurements.

#### 2.2.7. Fluorescence Spectroscopy

A Spectrofluorometer Fluorolog-3 Jobin Yvon-Spex (model GL3–21) was employed to record fluorescence spectra at room temperature. CUR measurements: Emission and excitation slits were both set at 2 nm, and the excitation wavelength was set at 405 nm. Pyrene measurements: Emission and excitation slits were both set at 2 nm, and the excitation wavelength was set at 335 nm. The I_1_/I_3_ ratio was obtained by dividing the intensity of the first peak (I_1_) by that of the third peak (I_3_). All the fluorescence measurements were recorded over a wavelength range of 350–700 nm.

#### 2.2.8. Cryogenic Transmission Electron Microscopy (Cryo-TEM)

Cryo-TEM images were obtained using a Tecnai F20 X TWIN microscope equipped with a field emission gun, operating at 200 kV. Images were captured using the Gatan Rio 16 CMOS 4k camera and processed with Gatan Microscopy Suite (GMS) version 3.31.2360.0 software. The process of preparing specimens involved the vitrification of aqueous solutions on grids covered with holey carbon film. As a first step, the grids were subjected to 15 s of activation using oxygen plasma via a Femto plasma cleaner. Cryo-samples were created by placing a droplet of the suspension onto the grid, blotting it with filter paper, and rapidly freezing it in liquid ethane using an automated blotting device called the Vitrobot Mark IV. The vitrified specimens were stored under liquid nitrogen until they could be analyzed at −178 °C using a cryo-TEM holder Gatan 626.

## 3. Results

### 3.1. SEC Analysis

**Figure 2:**
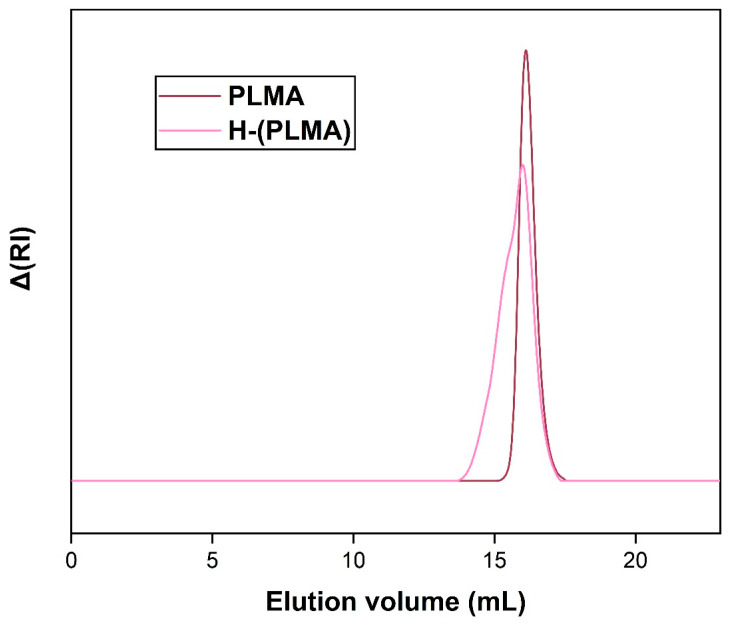
SEC curves of hyperbranched and linear PLMA in THF.

The SEC traces of polymers H-(PLMA) and PLMA are shown in Figure 2. The M_w_ of each homopolymer was 11,300 g mol^−1^ and 6200 g mol^−1^, with a polydispersity index (Ð) of 1.38 and 1.09, respectively (Table 1). It is typical for hyperbranched structures to have true weight-average molecular weights substantially higher than determined values by SEC, as linear polymer standards are utilized for the calibration of the instrument.

### 3.2. Neat Polymer Nanostructures Obtained after Nanoprecipitation

#### 3.2.1. Light Scattering Results

**Figure 3:**
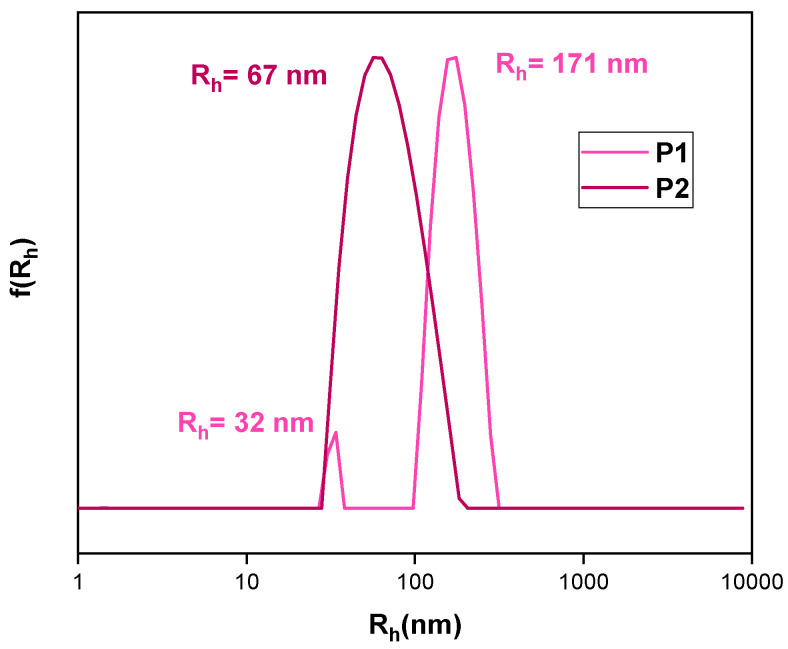
Size distributions from CONTIN analysis of neat nanoparticles. Concerning P1 and P2’s concentration in the organic phase, P1:P2 = 3. C_H-(PLMA)_ = 10^−4^ g/mL in water for injection.

The dimensions of candidate nanoparticle excipients affect multiple factors such as macrophages and/or kidney elimination, tumor permeability, cell uptake, etc. [39,40,41,42]. Hydrophobic interaction-driven self-assembly is a tried-and-true method based on the concept of the nanoprecipitation method. Macromolecules are dissolved in an organic solvent and quickly dispersed in an aqueous medium, which serves as the “bad solvent.” Agitation and thermal energy result in the formation of nanoparticles [43]. The size and form of these nanoparticles can be affected by a variety of factors, including the amount of homopolymer present in the organic phase. The remarkable achievement of establishing aqueous solubility of extremely hydrophobic moieties of H-(PLMA) is demonstrated in Figure 3. Diverse polymer concentrations in the organic phase were evaluated to guarantee replicability. P1 to P4 represent a different concentration of H-(PLMA) in THF with the same resulting aqueous concentration. The higher the number, the more concentrated the suspension (Table 2). 

Optimal results were obtained in the case of P2 (R_h_ = 67 nm, PDI = 0.152) as small nanoparticles with a narrow unimodal size distribution were produced. This can be attributed to the mild hydrophilicity the CTA agent –COOH end groups can offer. Mainly the formation of carboxylic acid groups and water molecule hydrogen bonds assist in the formation of a hydrophilic shell. Given their size, the first population of particles cannot be classified as unimers and are most likely unstable aggregates that aggregate further to form larger structures that were also observed as the second population group. Regarding P1, P2’s concentration in the organic phase can be expressed as P1:P2 = 3 (polymer concentration increases in each sample) after various quantities were tried. These results indicate that lowering the homopolymer concentration in the organic phase yields smaller nanoparticles.

Moreover, there is a slower transition to a hydrophobic environment. This transition is a very important timestamp in this method, the spontaneous emulsification process is known as the “Ouzo effect”. This term refers to the phenomenon that occurs when a water-insoluble entity, originally trans-anethol (in our case H-(PLMA)) is dissolved in alcohol, a water-miscible solvent (in our case THF), and spontaneously is dispersed in water giving a blurry white effect due to the formation of long-lived metastable oil droplets which scatter visible light. Consequently, in our case, the formation of nanoparticles is observed (ouzo region) [43]. Table 2 illustrates how large-mass nanoparticles are produced when the solvent/water ratio is increased. Furthermore, size dispersity decreases while the hydrodynamic radius appears to remain constant, supporting the idea of highly homogeneous and compact nanoparticles. Research has indicated that this parameter plays a critical role in the final size of nanoparticles in the following cases: poly(ethylene oxide)-block-polycaprolactone [39], mild hydrophobic homopolymers such as poly(D,L)-lactides [44] and polymethylmethacrylate [45] and hydrophilic homopolymers such as poly(vinyl acetate) [43]. These findings indicate that larger nanoparticles were produced as a result of higher polymer concentration in the solvent phase.

**Figure 4:**
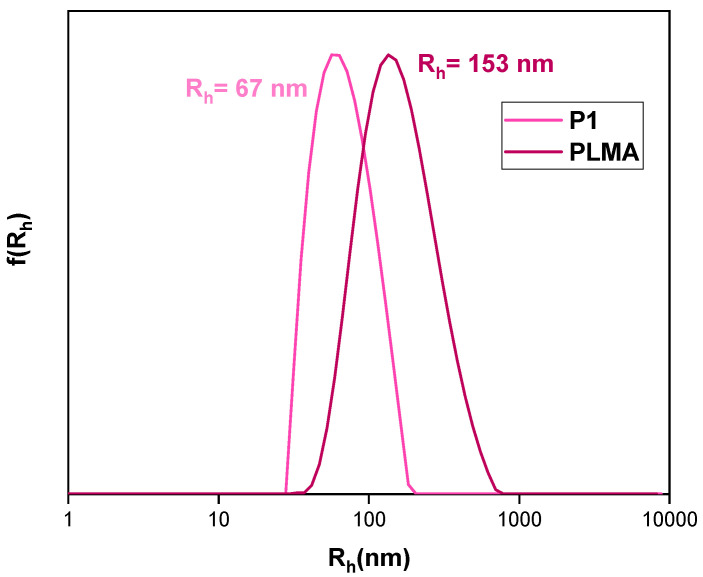
Size distributions from CONTIN analysis of neat nanoparticles. C_P1_ = 10^−4^ g/mL, C_PLMA_ = 5 × 10^−5^ in distilled water.

Linear PLMA prepared with identical conditions to P2 was utilized as a means to assess the influence of the branching agent. The resultant colloidal system differed significantly from its hyperbranched analog (Figure 4), necessitating dilution to estimate the structure’s size. Since the resultant sample was opaque white and above the range for DLS measurements as strongly scattered light, a 1:1 dilution rate with WFI was used. As illustrated in Table 2, much larger and rather heterogeneous particles are formed. These results indicate that the hyperbranched homopolymer is superior as each macromolecule incorporates numerous end carboxyl groups that contribute to the system’s hydrophilicity, which balances the lengthy hydrocarbon backbone’s hydrophobicity.

#### 3.2.2. Zeta Potential (ζp)

When nanoparticles are suspended in an aqueous medium, they acquire a net surface charge, which influences a variety of properties. Depending on the size of these nanostructures, agglomeration is prevented, and electrostatic repulsion occurs when the surface charge is strong. As a general rule, the zeta potential must be ≤ −30 mV or ≥ +30 mV to be regarded as stable throughout time. Additionally, the toxicity and cell adsorption aspects of nanoparticles are significantly influenced by this value [46,47,48,49] Table 3 displays the results; as expected, P1 had the lowest values and was the most unstable of the bunch, resulting in the aggregation of emergent structures. The real value is closer to zero when taking the standard deviation into account, thus providing the only positive number that can support the notion that the non-ionic LMA segments are on the surface of the structure. The presence of carboxyl groups on the end chains of each branch generates the negative ζp readings. The value difference between the linear and the hyperbranched counterpart amplifies the scenario of increasing solubility/stability of nanoparticles as the number of carboxyl groups increases. These results indicate that the bulk of these samples fit the definition of colloidally stable systems. Significant stability in biological media is correlated with increased zeta potential; the protein studies support this idea [50].

### 3.3. Cargo Loaded Nanostructures and Mixed Systems

#### 3.3.1. Pyrene Encapsulation

**Figure 5:**
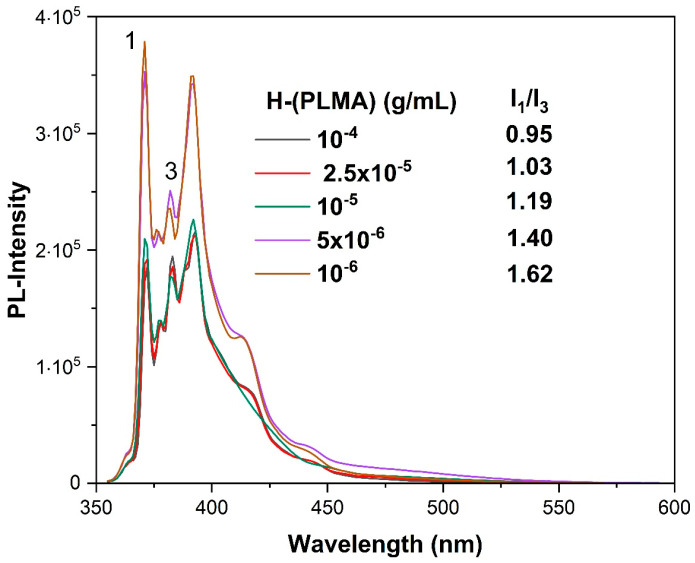
Fluorescence spectra of pyrene added in H-(PLMA) aqueous solution. Numbers 1 and 3 are arbitrary references to the corresponding peaks of pyrene PL spectrum.

To highlight this homopolymers’ great adaptability as a nanocarrier, a wide range of hydrophobic payload molecules were effectively encapsulated. Employing pyrene as a fluorescent probe allowed for the estimation of the loading capacity of this type of nanoparticle. The intensity ratio (I_1_/I_3_) of pyrene excitation spectra is a sensitive measure of the polarity of the microenvironment around pyrene [51]. Pyrene preferentially localizes in the hydrophobic domains of amphiphilic molecules due to its low aqueous solubility. Large values of this ratio indicate hydrophilic systems, whereas smaller values indicate hydrophobic systems. Samples were prepared by adding 1 mM pyrene solution in acetone at a 1 μL/mL ratio to the hyperbranched polymer solution. Polymer concentration ranged from 10^−4^–10^−6^ g/mL. Figure 5 displays the results. As the homopolymer concentration increased, the value of I_1_/I_3_ dropped since pyrene molecules accumulated in the hydrophobic microenvironment of the H-(PLMA) nanostructures. These results suggest that 10^−4^ g/mL is a suitable concentration for encapsulating hydrophobic payload. Studies verify that low values within the 1.0–1.3 range indicate a non-polar environment surrounding pyrene. In the range of 1.7–1.9, a more hydrophilic environment is confirmed [52].

#### 3.3.2. Mixed Systems with Tween 80 Surfactant

**Figure 6:**
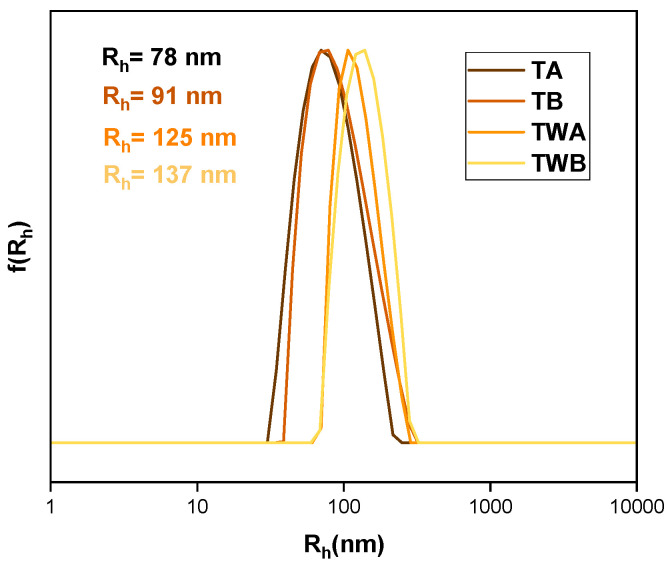
DLS size distributions from mixed Tween 80–H-(PLMA) aqueous solutions.

Tween 80 (Polysorbate 80) is a food-grade nonionic emulsifier frequently used in nanoformulations as a solubilizing agent, either as a stand-alone agency or a co-surfactant [53,54,55]. Mixed H-(PLMA)/Tween 80 systems were assessed using the “worst” performing formulation, P1, to maximize this surfactant’s potential. The resulting mixed systems were diluted by a factor of 3 for DLS measurements due to their large initial turbidity. Results can be found in Table 4 and Figure 6. Often, the predominant method is the dispersion of the surfactant in the aqueous solution [35]. It was observed that the method was not successful based on these findings. TWA and TWB nanoparticles had significantly larger mass and size. This could be because the surfactant struggled to reach the homopolymer in the aqueous medium. Conversely, when solubilized in a common organic solvent, the mixing was more effective. Additionally, the ζp results indicate that a looser configuration was formed as evidenced by a drop in values. This type of surfactant has a masking ability due to its non-ionic nature, which allows it to conceal the carboxyl groups of the homopolymer.

#### 3.3.3. Curcumin Encapsulation

##### Light Scattering Measurements

**Figure 7:**
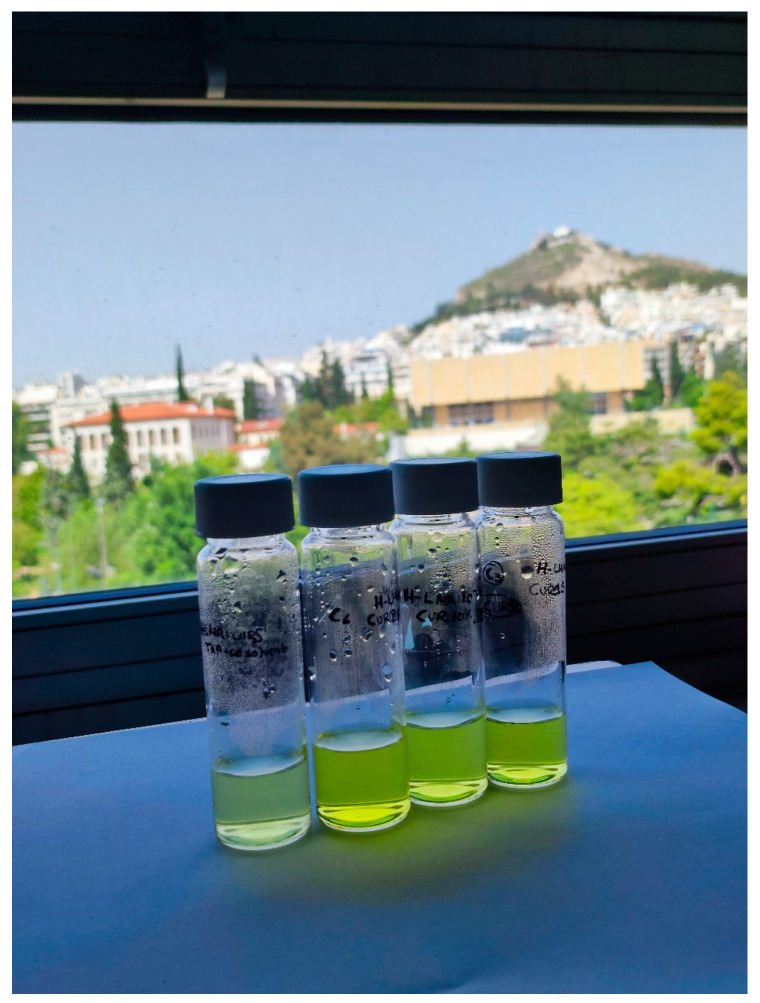
Curcumin-loaded nanoparticles. From left to right, CUR5, CUR8, CUR10, and CUR15 solutions.

**Figure 8:**
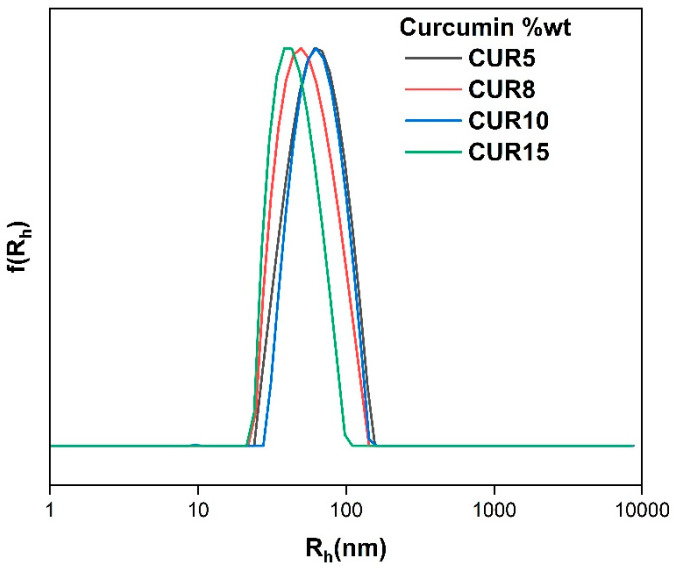
Size distributions of curcumin containing mixed nanostructures.

Turmeric, a well-known Indian spice with millennia of medicinal history, is the source of curcumin. This polyphenol has antioxidant, anti-inflammatory, antimicrobial, and anticarcinogenic properties [56]. However, these beneficial effects are lost due to various limitations, in vivo degradation, poor gut absorption, and very low aqueous solubility [57]. Thus, it is of the essence to find nanocarriers to boost the bioavailability of such hydrophobic cargo. Curcumin-loaded nanostructures were prepared at various weight ratios, namely 5 → 15 wt% with respect to polymer, (Figure 7, CURx refers to the weight percentage of curcumin) to determine the maximum curcumin loading in the polymer matrix and its effects on size, PDI, mass, and surface charge (Table 5). It is important to emphasize that each sample had satisfactory outcomes, especially in terms of the size and monodispersity of the emergent mixed structures (Figure 8). The structures exhibited R_h_ values within the range of 45–67 nm, accompanied by a PDI ranging from 0.107 to 0.153 . These narrow size distributions are akin to those of the blank structures and are found to be smaller than formulation P2, which served as the basis for each drug-loaded assembly. This can be explained by the hydrophobic drug being tightly packed in the core acting as a synergistic parameter enhancing the hydrophobic interactions. Unfortunately, there is not much information on hyperbranched hydrophobic macromolecules. However, in mixed systems with the help of surfactants, hydrophobic homopolymers like polycaprolactones have shown higher values than this study [58,59]. Similarly, in the case of mildly hydrophobic polylactide nanoparticles larger structures were produced [60]. The end carboxyl groups could form hydrogen bonds with the two phenolic –OH groups of curcumin, providing extra stability to the mixed aggregates. Nevertheless, the size of each formulation is well within the optimal range for biomedical applications. The surface charge is highly negative, as discussed in previous sections, it is due to the carboxyl end groups of the homopolymer. Considering these high values, these formulations can be classified as colloidally stable. Furthermore, positively charged nanoparticles are more likely to segregate in the liver, spleen, and lungs. Beyond that, serum proteins do not interact with neutral or mildly negatively charged nanoparticles, prolonging the half-life of the formulation [61].

##### Absorption and Emission Spectra Measurements

**Figure 9:**
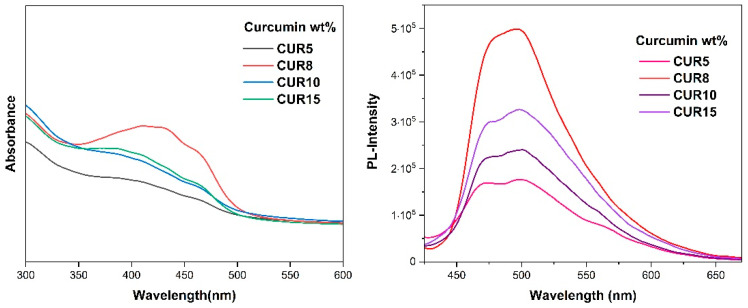
UV–Vis spectra of curcumin-loaded H-(PLMA) nanoparticles (left) and fluorescence spectra of curcumin-loaded nanoparticles (right).

Many medical conditions benefit substantially from early detection and intervention. Nanoformulations contribute greatly to the development of screening procedures that allow for the detection of several irregularities at microscopic levels. Therefore, it makes sense for diagnostic methods such as fluorescence imaging (FI) to make use of this possibility provided by nanoparticles [62,63,64,65,66]. As previously mentioned, curcumin has poor aqueous solubility, making it unusable as a lone molecule in bioimaging applications. The same benefits are amplified and preserved over time under the same circumstances with the simple addition of an amphiphilic solubilizer. The absorption spectra of these systems were studied by keeping the concentration of the polymer consistently fixed at 10^−4^ g/mL and adjusting the concentration of the dye from 0 to 15 % wt. Curcumin, although insoluble in water, displays a broad band in the area of 430 nm and a shoulder band in the range of 355 nm in high concentrations at neutral pH. The first stems from π → π* transitions (pi-bonding orbital (π) to antibonding pi orbital (π*)) of lowest energy and the shoulder band is assigned to the transitions π → π* of the feruloyl unit [67,68]. Based on these findings, the drug was adequately encapsulated on each occasion; by increasing the concentration of curcumin we see an increase in absorbance. The exemption is CUR8 which suggests that a favorable configuration is achieved that permits a higher encapsulation ability and/or a looser structure is formed allowing for less quenching (Figure 9). Minimal shifts were recorded, as well as noticeable changes in the absorbance intensity.

The use of fluorescent bioimaging in medicine has become more mainstream. Drug dye labeling in tumor treatment has become routine [69]. To explore the capabilities of these systems, the same samples were tested. This dye displays a broad maximum of practically zero value of fluorescence intensity at 551 nm in water [70]. In all cases, a blue shift to ~500 nm occurs, attributed to hydrophobic interactions between curcumin and the hydrophobic LMA moieties (Figure 9). It has been shown that the hypsochromic shift correlates with the hydrophobicity of the polymer and in terms of the spectral range of the photoluminescence, emitted light at 500 nm signifies a highly hydrophobic environment [71]. Overall, curcumin’s fluorescence properties were maintained, and its water solubility was achieved in the form of loaded nanostructures.

#### 3.3.4. IR-1048 Dye Encapsulation

**Figure 10:**
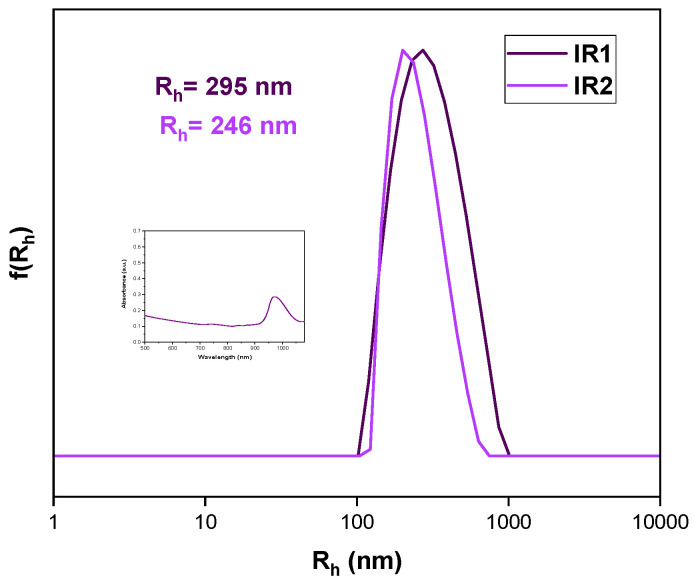
Comparative graph of size distributions of IR-1048 dye-loaded nanoparticles. Dispersion method: Nanoprecipitation protocol THF–ethanol, weight content of dye in respect to polymer: IR2 = 1% IR1 = 3% wt. in respect to H-(PLMA).

IR-1048 is a NIR-II hydrophobic dye. The second near-infrared window (1000–1700 nm) light exhibits less scattering than NIR-I light, which leads to deeper penetration into tumor tissue [72]. In bioimaging applications, it is essential to conjugate or encapsulate polymethine molecules (IR-1048) into biocompatible polymers due to their insolubility in water. The dye was effectively complexed with a hyperbranched homopolymer to address its hydrophobic nature and enhance its bioavailability. Figure 10 illustrates a slight shift to a shorter wavelength after encapsulation, compared to when IR-1048 is in its free form [72,73]. The dye was dissolved in ethanol due to its higher solubility and then mixed with the homopolymer, which was solubilized in THF. We calculated weight content based on the homopolymer concentration (10^−4^ g/mL). Nanoprecipitation was performed as described in previous sections. The largest values were observed after encapsulation, with a highly acceptable polydispersity (0.207, 0.225). The ζp values were −16.3 mV and −18.6 mV, respectively. These results were in line with our expectations given the structure of the cyanine dye. However, there is limited published data on the complexation of polymers and such dyes, making this a great starting point for further research.

#### 3.3.5. Cryo-TEM Analysis

**Figure 11:**
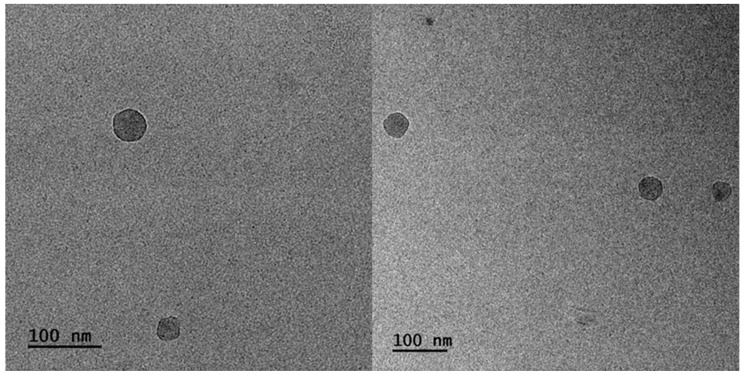
Cryo-TEM images of H-(PLMA) nanoparticles in water.

**Figure 12:**
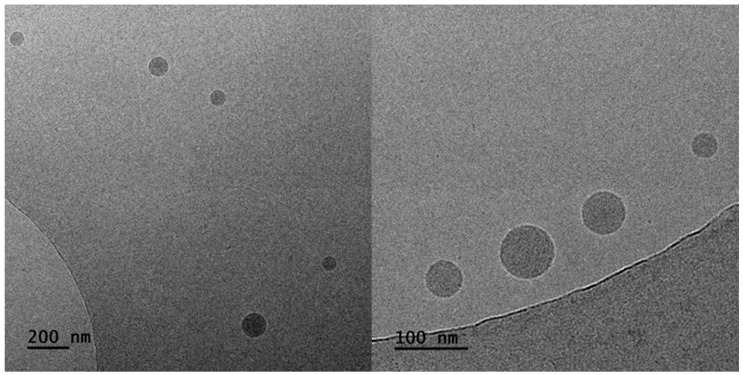
Cryo-TEM images of CUR8 mixed nanoparticles.

The morphology and the internal structure of particles composed of the H-(PLMA) homopolymers with carboxyl end groups were determined by Cryo-TEM. Representative Cryo-TEM images of H-(PLMA) nanoparticles in water are provided in Figure 11. The morphology of the nanoparticles was observed to be close to spherical. The images suggest a highly compact structure. The hydrophilic layer is not visible something common for this form of microscopy [74]. The nanostructures were found to be 15–60 nm in diameter. TEM images of the nanoparticles suggested they have smaller sizes than those measured by DLS. This is common because DLS provides information on the structures in a liquid state where surface charge and other factors play a pivotal role in agglomeration and TEM in a dry state. In Figure 12, it is observed that the curcumin-loaded sample CUR8 appears considerably more spherical compared to the neat nanostructures. The diameter of the structures is noticeably larger, ranging from 30–90 nm. These results suggest that the incorporation of the hydrophobic drug has a positive impact on the overall uniformity and structural properties of the system.

### 3.4. Suspension of Nanoparticles in Fetal Bovine Serum

**Figure 13:**
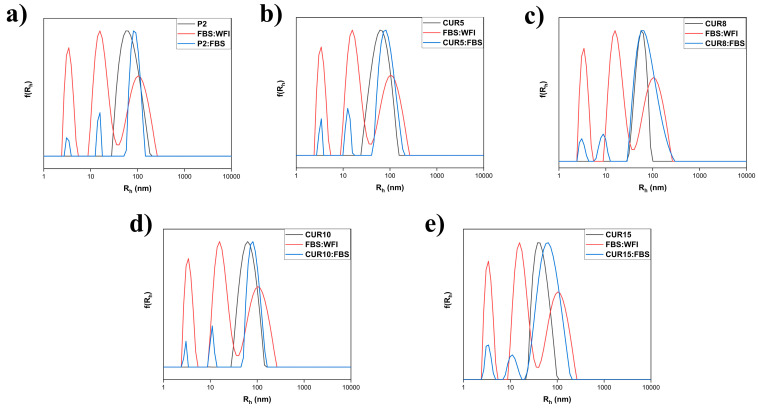
Comparative size distributions of P2 (**a**) and CUR5–CUR15 (**b**–**e**) preparations in FBS.

The biocompatibility of the newly developed mixed nanosystems was assessed. This was conducted by subjecting them to fetal bovine serum to gauge the potential formation of a protein corona, which could subsequently impact the surface chemistry of the nanoparticles. The FBS was diluted with WFI to achieve a 1:1 volume ratio before being introduced to the nanomaterials. The experiment revealed that minimal interaction was observed between the proteins and the polymer nanostructures, indicating a noteworthy aspect of the nanomaterials’ behavior in a simulated biological environment (Table S1 and Figure 13).

### 3.5. Stability Studies

It is essential to keep in mind that the stability of a pharmaceutical formulation is vital for safeguarding and ensuring the effectiveness of a drug over time. Stability can be described in terms of thermodynamic and kinetic stability. Thermodynamic stability is attained when the polymer concentration exceeds the critical aggregation concentration (CAC) while kinetic stability is linked to the presence of an energy barrier that prevents agglomeration [75]. The stability of the emergent structures was investigated by storing each formulation at ambient temperature and the data was examined over a period of time to mimic conventional drug conditions The same stock solution was used to avoid batch-to-batch variability, and it was kept airtight to avoid contamination. The DLS graphs list the various timeframes in which samples were measured. Structures loaded with curcumin demonstrated exceptional durability over time, making them ideal for applications in nanomedicine (refer to Figure S4).

## 4. Conclusions

To the best of our knowledge, very little data has been published about hyperbranched ionomer homopolymer-based nanostructures in aqueous media through a nanoprecipitation process like the ones reported in this study. The emergent nanostructures bear long alkyl side chains and carboxylic end groups, the former making them suitable for hosting hydrophobic cargo and the latter for the modulation of their aqueous solubility. Comparison with linear PLMA bearing only one –COOH group shows that the increased number of carboxylate groups enhances polymer solubility and results in the formation of smaller particles. The concentration of the homopolymer in the media also determines the size of the nanoparticles formed. As proof of concept of the biomedical applications of H-(PLMA) as carriers, the highly insoluble drug curcumin, and the highly hydrophobic dye IR-1048 were successfully encapsulated. These results suggest that this formulation can provide a useful alternative as a bioimaging excipient which could bypass the limitations of current formulations. This work serves as a useful starting point for discussion and additional research on end group-driven solubility of hydrophobic homopolymers with branched architectures in water via the nanoprecipitation method.

## Figures and Tables

**Table 1:** Characterization of the Hyperbranched and Linear LMA Homopolymers.

Sample	M_w_ (g/mol) ^[a]^	M_w_/M_n_^[a]^
H-(PLMA)	11,300	1.38
PLMA	6200	1.09

^[a]^ M_w_ stands for weight average molecular weight. M_n_ stands for number average molecular weight.

**Table 2:** DLS data for the prepared nanoparticles.

Sample Code	Intesity_90°_ (a.u.)	R_h_ (nm) ^a^	PDI (Polydispersity Index)
P1	1952	32 171	0.295
P2	22,419	67	0.152
P3	34,900	71	0.146
P4	62,000	75	0.108
PLMA *	6300	153	0.351

* A 1:1 dilution was needed for the linear polymer. ^a^ Based on Contin analysis. The P1 solution contained two populations of nanoparticles.

**Table 3:** Zeta potential values of neat nanostructures.

Sample	Zeta Potential (mV)
P1	+4.99
P2	−29.6
P3	−28.2
P4	−31.2
PLMA	−22.3

**Table 4:** DLS results of Tween 80–H-(PLMA) mixed systems.

Sample	Intensity_90˚_ (a.u.)	PDI	R_h_ (nm)	Z-Potential (mV)
TA	3560	0.182	78	−19.9
TB	5950	0.159	91	−21.9
TWA	12,258	0.113	125	−31.3
TWB	7200	0.112	137	−33.9

**Table 5:** DLS results of curcumin containing mixed nanostructures.

Sample	Intensity_90°_ (a.u.)	PDI	R_h_ (nm)	Ζ-Potential (mV)
CUR5	21,064	0.153	62	−23.2
CUR8	19,000	0.15	54	−29.2
CUR10	32,500	0.122	67	−34.2
CUR15	14,484	0.107	45	−34.4

## Data Availability

The original contributions presented in the study are included in the article/Supplementary Materials, further inquiries can be directed to the corresponding author.

## Supplementary Materials

The following supporting information can be downloaded at https://www.mdpi.com/article/10.3390/polym16152166/s1: Figure S1: Superposition of ^1^H–NMR spectra of H-(PLMA) (red) and PLMA (light blue) in CDCl_3_; Figure S2: FT–IR spectra of H-(PLMA) (left) and PLMA (right); Figure S3: P3 and P4 size distributions; Figure S4: DLS stability data at 50 day timeframe, for CUR5, CUR8, CUR10, CUR15 preparations (from top left to right); Figure S5: H-(PLMA) UV–Vis spectrum in water; Table S1: DLS data for FBS suspensions of nanoparticles; and Table S2: Zeta potential values-stability studies.
