# Supplementary material for: Self-Assembly of Hydrophobic Hyperbranched PLMA Homopolymer with –COOH End Groups as Effective Nanocarriers for Bioimaging Applications
Source: Polymers (Basel). 2024 Jul 30;16(15):2166. doi: 10.3390/polym16152166 (PMC11314538; doi:10.3390/polym16152166)
Supplement: Supplementary file 1 [file polymers-16-02166-s001.zip › polymers-3102347-supplementary.pdf]

Supporting information

Self-Assembly of Hydrophobic Hyperbranched PLMA Homopolymer with –COOH End Groups as Effective Nanocarriers for Bioimaging Applications

Angelica Maria Gerardos [1,2], Aleksander Foryś [3], Barbara Trzebicka, [3] and Stergios Pispas [1,*]

[1] Theoretical and Physical Chemistry Institute, National Hellenic Research Foundation, 48 Vassileos Constantinou Avenue, 11635 Athens, Greece; amgerar@eie.gr (A.M.G.); pispas@eie.gr (S.P.)
[2] Department of Chemistry, National and Kapodistrian University of Athens, Panepistimiopolis, Zografou, 15771 Athens, Greece
[3] Centre of Polymer and Carbon Materials, Polish Academy of Sciences, 34 ul. M. Curie-Skłodowskiej, 41-819 Zabrze, Poland; aforys@cmpw-pan.pl (A.F.); btrzebicka@cmpw-pan.pl (B.T.)
* Correspondence: pispas@eie.gr

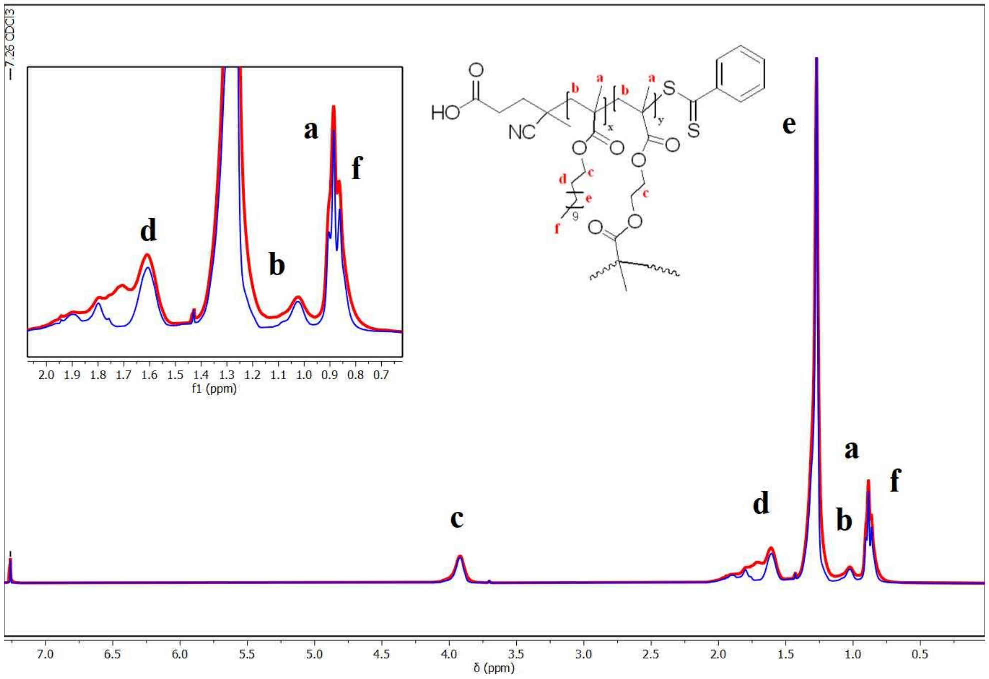

Figure S1. Superposition of ^{1}H-NMR spectra of H-(PLMA) (red) and PLMA (light blue) in $CDCl_3$.

The ^{1}H-NMR spectra of hyperbranched and linear PLMA are shown above (Figure S1). Characteristic peaks such as those of -CH_2- groups of LMA at 1.27 ppm and EGDMA/LMA at 3.92 ppm corroborate the successful synthesis. No significant differences arise from the use of the branching agent, except for a steeper spectrum texture in the region 1.70-1.95 ppm.

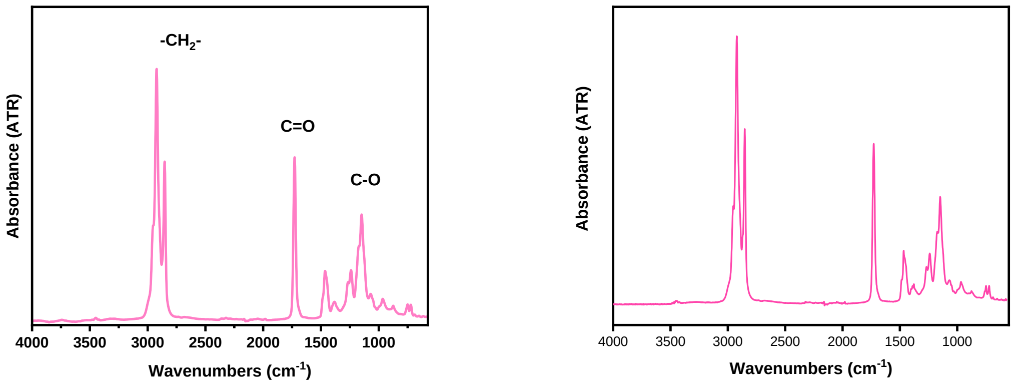

Figure S2. FT-IR spectra of H-(PLMA) (left) and PLMA (right).

ATR FT-IR using the attenuated total reflectance (ATR) method was performed on the gel-like neat polymer. The main features observed were the strong $-CH_2-$ stretching vibration of alkyl chains at 2850-2990 cm^{-1}, the ester carbonyl stretching centered at 1726 cm^{-1} and the C-O stretching at 1147 cm^{-1}. No significant changes were observed upon the use of the branching agent, leading to a nearly identical spectral footprint.

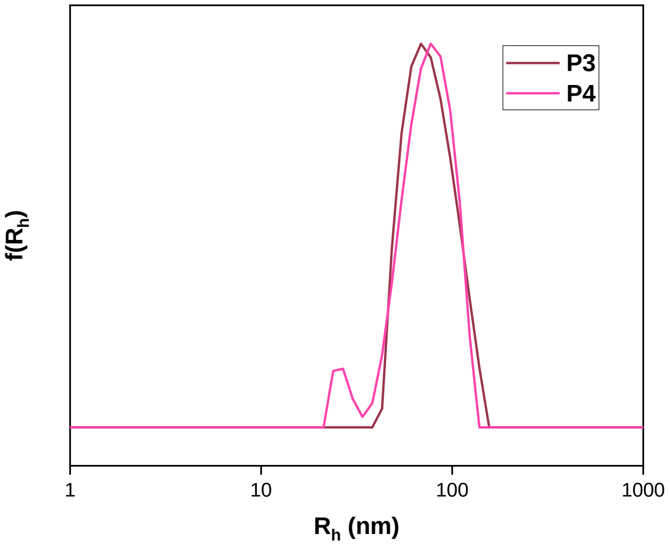

Figure S3: P3 and P4 size distributions.

Table S1. DLS data for FBS suspensions of nanoparticles.

Formulation	Intensity (kHz)	PDI	R_h(nm)
FBS:WFI	11935	0.521	2<br>19<br>84
CUR5/FBS:WFI	17097	0.458	13<br>86
CUR8/FBS:WFI	17968	0.471	8<br>73
CUR10/FBS:WFI	19226	0.452	2<br>19<br>84
CUR15/FBS:WFI	14000	0.452	12<br>65
P2/FBS:WFI	19387	0.46	4<br>15<br>85

Table S2. Zeta potential values-Stability studies

SAMPLE	ζ potential (mV)
CUR5	-32.6
CUR8	-37.1
CUR10	-40.9
CUR15	-36.5
P2	-29.6

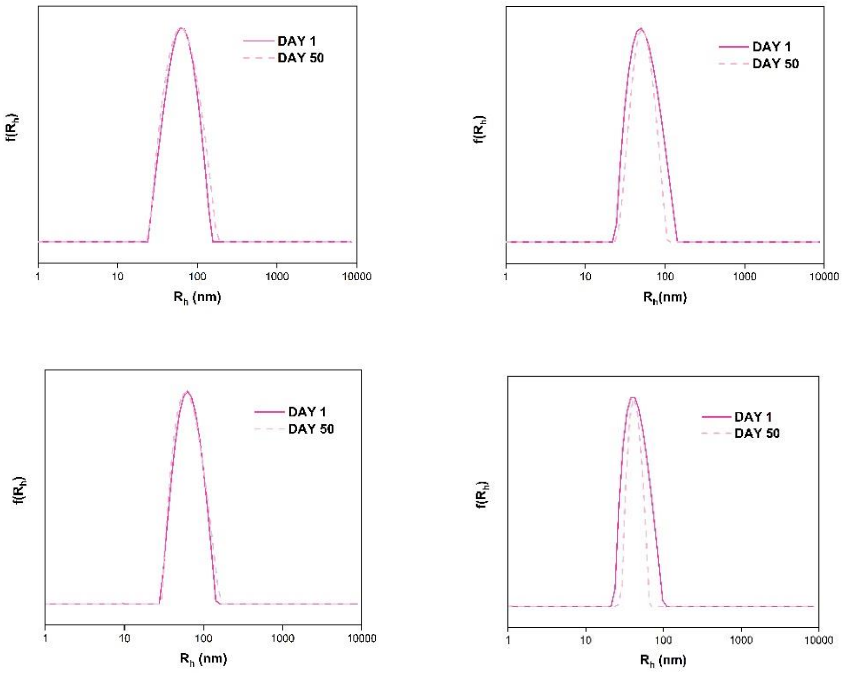

Figure S4. DLS stability data at 50-day timeframe, for CUR5, CUR8, CUR10, CUR15 preparations. (From top left to right)

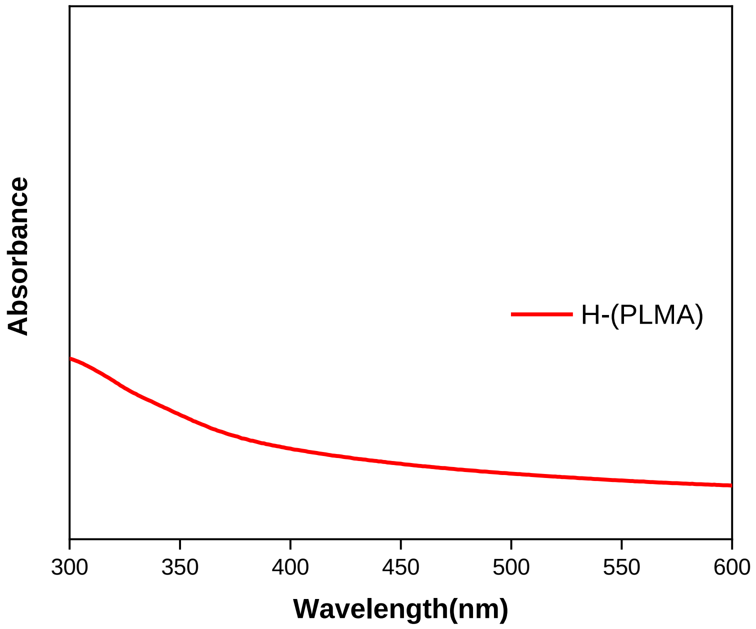

Figure S5. H-(PLMA) UV-Vis spectrum in water.
